# COVID-19 changes medical education in Italy: will other countries follow?

**DOI:** 10.1136/postgradmedj-2020-137876

**Published:** 2020-05-13

**Authors:** Pierfrancesco Lapolla, Andrea Mingoli

**Affiliations:** Department of Surgery P. Valdoni, Policlinico Umberto I, Sapienza University of Rome, Roma, Italy; Department of Surgery P. Valdoni, Policlinico Umberto I, Sapienza University of Rome, Roma, Italy

The severe acute respiratory syndrome coronavirus 2 (SARS-CoV-2) pandemic is bringing the world to its knees with over one million cases by 8 April 2020.^1^ Several governments have responded by locking down entire countries with dramatic repercussions for all sectors of society. Since 21 February, the total number of cases in Italy has followed an exponential trend with 139 422 (including 13 522 healthcare workers) by April 8, which is four times higher than 3 weeks before (35 713 confirmed cases on March 18).^1^ Among the currently active cases (n=95 262), 28 485 (29.9 %) are hospitalised and 3693 (3.87 %) in intensive care units (ICUs).^1^ Italy has overtaken China with the most SARS-CoV-2 deaths, reporting 17 669 fatalities,^1^ a fatality rate of 12.6% (of confirmed cases, table 1).

**Table 1:** Estimated increase in hospital doctor workforce in the COVID-19 outbreak in Italy shown per region

Regions of italy	Final year medical students (n=9640)^1^	Hospital medical doctors (n=92 950)^5^	Estimated increase % in medical doctor workforce (10.3%)	COVID-19 current positive cases (n=95 262)^1^	Hospitalised patients (n=28 485)^1^ 29.9%	Patients admitted to intensive care unit (n=3693)^1^ 3.87%	Deaths (n=17 669)^1^	Case fatality rate (12.6%)*
Lombardy	795	16 114	4.9	28 545	11 719	1257	9722	18.2
Emilia Romagna	636	6844	9.3	1311	3769	361	2234	12.3
Piedmont	537	7530	7.1	10 989	3493	423	1378	9.9
Veneto	427	7347	5.8	10 171	1554	285	736	59.3
Liguria	112	3172	3.5	3245	1109	153	654	13.3
Marche	96	2412	4.0	3562	974	133	652	13.4
Tuscany	533	6921	7.7	5557	1066	260	392	6.1
Trento	0	915	0.0	194	354	77	255	9.8
Lazio	1403	8337	16.8	3448	1241	196	244	5.7
Campania	1789	6390	28.0	2859	608	97	221	6.8
Apulia	510	6094	8.4	2238	639	90	219	8.3
Bolzano	0	945	0.0	1281	268	65	183	10.0
Abruzzo	518	2044	25.3	1534	331	62	179	9.6
Friuli V.G.	87	2077	4.2	1415	162	41	169	7.6
Sicily	987	6786	14.5	1893	563	65	133	6.2
Aosta Valley	0	288	0.0	606	120	20	102	12.0
Calabria	144	2446	5.9	755	170	15	60	7.0
Sardinia	359	3318	10.8	840	112	31	59	6.1
Umbria	633	1632	38.8	823	155	41	50	3.9
Basilicata	0	913	0.0	270	48	17	14	4.7
Molise	74	425	17.4	181	30	4	13	5.8

Data are from the Italian Ministry of Health, Italian Ministry of Education University and Research.

*Based on the total count of 139 422 confirmed cases on 8 April 2020, 17h00 UTC+1.

The immediate and urgent demand for more doctors has led the Italian government to take unprecedented measures. On 17 March 2020, the Council of Ministers passed the Cura Italia decree which changed the rules of Italian medical board examinations.^2^ As a result, almost 10 000 Italian medical students from all medical schools will be fast-tracked into the healthcare system after graduation, without sitting the postgraduate examination which concludes the practical training.^3^ This change is permanent.

In the UK, the Medical Schools Council has suggested the possibility of releasing final-year medical students, even before the conclusion of their clinical examinations, to be provisionally registered by the General Medical Council in order to help the healthcare system to cope with the developing crisis.

Before Cura Italia, the Italian medical licence required postgraduate training and an exam. The new decree permanently makes the medical degree fully qualifying. Licence training will now take place in the years before graduation. Therefore, it is estimated that 9640 newly graduated medical doctors will be qualified earlier to join the healthcare system.^4^ This could see a rapid 10.3% increase in hospital doctors, supporting departments and ICU dedicated to COVID-19 treatment in all regions. For instance, Lombardy, with 9722 deaths,^1^ a 18.2% case fatality rate of confirmed cases, might benefit from a 4.9% increase in doctor numbers; Emilia Romagna, the second most affected region, could have an increase of 9.3% (table 1).^5^ Also, these graduates might cover roles in less front-line areas from which medical personnel have transferred to acute care. Italy is, therefore, the first country to respond formally to the COVID-19 emergency by permanently changing the medical board examinations and altering the curriculum.

On the one hand, the decree shortens the licensing process by about 9 months,^3^ a crucial time for the immediate movement of thousands of new doctors into the workforce for the COVID-19 emergency. On the other hand, a shift from medical school to clinical work without a transition period might put graduates at a greater risk of work-related stress. Furthermore, without a written examination, more emphasis would be on the assessment of the practical training during medical school.

The provisions adopted by the Italian government regarding the permanent change of the rules for the medical board examination and the consequent fast-tracking of doctors will affect medical education for future students and augment the healthcare workforce to improve care during the current crisis. Consequently, the new Italian model might be considered by other countries. However, transparency and clear guidelines for newly qualified doctors and precise human resource planning of the new healthcare workforce are essential to safeguard patient safety in one of the gravest challenges of our time.
